# The Impact of an Antimicrobial Stewardship Clinical Pharmacy Specialist on Antimicrobial Days of Therapy through Education Driven Policies, Procedures, and Interventions

**DOI:** 10.3390/pharmacy11050137

**Published:** 2023-08-30

**Authors:** Yolanda G. Martinez, MaiCuc Tran, Thomas Roduta, Susan Lam, Todd Price, Stefanie Stramel

**Affiliations:** 1 Memorial Hermann Memorial City Medical Center, Houston, TX 77024, USAthomas.roduta@memorialhermann.org (T.R.); tprice@priceid.com (T.P.); 2 Memorial Hermann Health System, Houston, TX 77024, USA; susan.lam@memorialhermann.org

**Keywords:** infectious diseases, antimicrobial stewardship, interventions

## Abstract

The primary goal of antimicrobial stewardship is to improve patient outcomes and minimize the consequences of antibiotic use. Prospective audit and feedback cannot always be performed by an antimicrobial stewardship program member which is where policies, procedures and education can aid interventions. The purpose of this study was to evaluate the impact on antimicrobial days of therapy due to a dedicated clinical pharmacy specialist primarily responsible for developing policies and procedures and providing education. A pre-intervention and post-intervention retrospective analysis of antimicrobial days of therapy from September 2019–May 2020 and July 2020–March 2021 was performed. Inclusion criteria consisted of adults receiving IV vancomycin, azithromycin, meropenem, ciprofloxacin, and/or levofloxacin. Excluded criteria consisted of documented interventions that were not related to implemented policies and procedures or performed education and patients receiving antimicrobial surgical prophylaxis. The primary outcome was antimicrobial days of therapy. An average of 3.47 ± 2.46 days (pre-intervention, *n* = 203) and 3.21 ± 2.52 days (post-intervention, *n* = 203) were observed for the primary outcome (*p* < 0.04182). Pharmacists performed 75 interventions pre-intervention and 102 interventions post-intervention (*p* = 0.0092). The implementation of a dedicated antimicrobial stewardship clinical pharmacy specialist responsible for developing policies, procedures, and education successfully reduced antimicrobial days of therapy and documented interventions.

## 1. Introduction

Antimicrobial stewardship programs optimize prescribing to help improve patient care, reduce hospital costs, and aim to decrease the development of antimicrobial resistance and the incidence of multidrug-resistant organisms (MDROs) [1].

The primary goal of antimicrobial stewardship is to improve patient outcomes and to minimize the consequences of antibiotic use [2]. However, this can be difficult as antimicrobial stewardship strategies vary based on available resources and level of care, including intravenous to oral conversions, prospective audit and feedback, dose optimization, implementation of rapid diagnostic testing, antibiotic use pre-authorization, etc. [3]. The strategy of prospective audit and feedback generally consists of case review by an antimicrobial stewardship program member and feedback recommendations if antibiotics are inappropriate or can be de-escalated [1]. The core of this strategy is to assess the appropriateness of antibiotics, the correct dosing of the antibiotic, and the appropriate duration of antibiotics [4]. Additionally, assessment of the duration of antibiotics is an important part of this strategy. In a study conducted by Teshome and colleagues, many infections treated with shorter durations of antibiotics showed that each additional day of unnecessary antipseudomonal β-lactam antibiotics was associated with an increased risk of developing new antimicrobial resistance within 60 days of initiating the antibiotic [5]. It was also found that each additional day of unnecessary cefepime use could result in an 8% increased risk of antimicrobial resistance [5]. These results helped to reinforce guideline recommendations of using the shortest effective antibiotic durations when treating hospital-acquired and ventilator-associated pneumonia [5,6,7].

One noted weakness of prospective audit and feedback is that it is very labor-intensive and also involves physician education [1]. Therefore, policies, procedures, and education are often utilized to increase antimicrobial stewardship practices across the hospital [1]. These methods allow for larger numbers of patients to receive the same intervention (e.g., shorter durations of therapy) without direct antimicrobial stewardship team member involvement. Prospective audit and feedback literature is readily available; however, stewardship efforts regarding policy and procedure development, implementation, and education has been sparsely evaluated [1]. Thus, primary and secondary observations would provide further insight into the benefit of a dedicated antimicrobial clinical pharmacy specialist on this aspect of antimicrobial stewardship.

Interventions, duration or otherwise, are necessary as antimicrobial resistance, as stated by the World Health Organization (WHO), is a public health problem worldwide [8]. In addition, the 2019 U.S. Centers for Disease Control and Prevention (CDC) report stated that over 2.8 million infections and 35,000 deaths every year are associated with antimicrobial resistance [8]. Two major issues in this area are the inappropriate use of antibiotics and MDROs [1,9,10]. As briefly mentioned, the inappropriate use of antibiotics, such as continuing the use of broad-spectrum antibiotics after susceptibilities have returned, can lead to these medications inducing resistance mechanisms or allowing for the opportunity of the development of these resistant organisms [1,8,10]. Additionally, estimates quoted that around 20–50% of prescribed antibiotics in the acute care hospital setting in the United States are unnecessary or inappropriate [8]. These inappropriate practices lead to unnecessary antimicrobial days of therapy, and the use of inappropriate or unnecessary antibiotics can result in bacterial cultures with future bacterial mutations (as previously described), ultimately rendering antibiotics ineffective [1,8,10].

A barrier to the strategy of prospective audit and feedback is that some physicians remain concerned about the evidence behind these recommendations, especially if the patient was not seen or examined directly by a member of the antimicrobial stewardship program [3]. Also, barriers to the intervention being performed by a pharmacy team member at the bedside are not readily known. Recommendations to discontinue therapy are variably accepted and are not uncommon, and if they are declined, they may result in prolonged antimicrobial use for the patient [1,6].

Current antibiotic stewardship program (ASP) literature recommends one ASP clinical pharmacist FTE (full-time employee) for every 100–250 occupied beds [5]. This highlights the importance of the antimicrobial stewardship role in education, policies, and procedures to all pharmacists not solely dedicated to infectious diseases (ID) [11]. A study by Heil and colleagues showed that prospective audit and feedback interventions decreased length of stay (LOS), regardless of whether the intervention was made by an antimicrobial stewardship dedicated pharmacist or a non-dedicated pharmacist with access to an antimicrobial stewardship pharmacist [11]. To our knowledge, there were no currently published studies evaluating the effects of antimicrobial stewardship education, policies, and procedures on pharmacist intervention prior to this study [1,2,3,12,13]. Thus, antimicrobial days of therapy, from this perspective, has also not been reviewed. The Society for Infectious Disease Pharmacists (SIDP) states that every pharmacist plays an important role in antimicrobial stewardship, regardless of whether their role is mainly dedicated to infectious diseases [13]. Due to antimicrobial resistance being a global issue, every pharmacist has a role in antimicrobial stewardship in order to make an attempted impact on reducing antimicrobial resistance [13]. In addition, CDC core elements recommend dedicated time to be provided to stewardship activities such as those that have been evaluated herein [14].

Utilized metrics for these identified outcomes of the antimicrobial stewardship team include antibiotic utilization, patient outcomes, process, and cost measures [4]. Specifically, days of therapy is a highly utilized standard metric of antimicrobial measures stewardship in the United States [4]. Therefore, the included antibiotics in this study—vancomycin, azithromycin, meropenem, ciprofloxacin, and levofloxacin—were assessed using days of therapy (DOT). Identified antibiotics were included because they represented antibiotics in which a policy, procedure, or education was implemented by a dedicated antimicrobial stewardship pharmacist.

Use and associated risks were driving forces for developed and/or implemented policies, procedures, and education. Fluoroquinolone use has been associated with an increased risk of antibiotic resistance, serious adverse effects, and *Clostridium difficile* infection [15]. In this community hospital, the fluoroquinolone antibiogram has shown consistently poor susceptibility levels. Vancomycin is judiciously used (albeit not always appropriately) for methicillin-resistant staphylococcus aureus (MRSA) infections (as they are a known elevated burden in healthcare and a concern worldwide), and recent guidelines indicate this as the drug of choice [16]. Specifically, the re-education of MRSA PCR nares was completed due to inconsistent PCR order placement for patients receiving vancomycin. Also, the vancomycin policy was broadened to include the placement of orders for patients with acute bacterial skin and skin structure infections (ABSSSI) as opposed to the previous approach in which only pneumonia patients received orders. Noted azithromycin literature has recently shown it to be efficacious when used for a course of 3 days vs. the standard 5-day prescribed courses for patients with atypical pneumonia [17]. Thus, an azithromycin protocol was implemented as part of antimicrobial stewardship efforts to decrease barriers to discharge. Lastly, meropenem use is typically recommended for reserved use in more serious infections with suspected or documented drug resistant organisms, and a medication utilization evaluation showed areas of opportunity to guide appropriate use [18]. All antibiotics carry significant risks or side effects if used inappropriately [16,17,19].

The objective of this study is to evaluate the impact of a dedicated clinical pharmacy specialist with primary responsibilities of developing policies, procedures, and education on antimicrobial days of therapy. This data contributes, to the best of our knowledge, findings that are the first of its kind.

## 2. Materials and Methods

### 2.1. Study Design

This study was a pre-intervention and post-intervention retrospective, observational study at a single center community care hospital in Houston, Texas. Antimicrobial stewardship interventions via prospective audit and feedback were performed with the assistance of real-time alerts provided by outside antimicrobial stewardship monitoring software used to identify patients for selected review by ASP personnel. Patients were included if they were adults (age ≥ 18 years) with antimicrobial consumption of selected antibiotics, including vancomycin, azithromycin, meropenem, ciprofloxacin, and levofloxacin, for time periods from September 2019–May 2020 and July 2020–March 2021. Patients were excluded if they had documented interventions that did not involve selected antibiotics, documented interventions that were not related to policies and procedures or performed education, oral vancomycin doses, or antimicrobial surgical prophylaxis. The primary outcome was antimicrobial days of therapy for vancomycin, azithromycin, meropenem, ciprofloxacin, and levofloxacin. The secondary outcomes included the review of documented interventions and associated cost avoidance.

Antimicrobial utilization was collected as days of therapy (DOT). The policies, procedures, and education evaluated in this study included: the implementation of vancomycin methicillin-resistant Staphylococcus aureus (MRSA) PCR nares re-education for pneumonia and education on policy inclusion of ABSSSI as an indication, vancomycin area under the curve (AUC) Bayesian dosing platform education with adoptive dosing per pharmacy protocol (whereas previous practices were per consult), fluoroquinolone medication use evaluation (MUE) results education in preparation for the implementation of restriction criteria, protocolized auto-interchange criteria for three- to five-day azithromycin therapy for patients who met selected criteria, and meropenem MUE education results also in preparation for restriction criteria. Education was delivered via a variety of platforms ranging from face-to-face feedback and memos to in-person meetings that were recorded as videos with follow-up competency-based questions. However, most education was performed via live education sessions that were recorded and made available with competency-based questions. These policies, procedures, and education were chosen for evaluation due to implementation and/or education during the post-intervention period. Interventions for these policies, procedures, and education were voluntary and independently documented by any member of the pharmacy department. The associated pharmacy department structure at this community hospital included pharmacy clinical specialists who were available Monday–Friday and 24/7 on-call in specialized areas, such as infectious diseases, internal medicine, pediatrics, intensive care unit (ICU), and emergency medicine. Decentralized pharmacists in this community hospital were available Monday–Sunday with decreased weekend hours, and centralized pharmacists were available 24/7.

### 2.2. Data Collection

All data were collected from medical charts, and documented interventions were obtained via a third-party, integrated stewardship electronic medical surveillance tool. Data collected included antimicrobial days of therapy for selected antimicrobials previously described, antimicrobial consumption, length of stay, antimicrobial stewardship documented interventions, cost savings associated with documented interventions, gender (female/male), patient age, antibiotic allergies and reactions, and hospital service (Table 1).

### 2.3. Statistical Analysis

Parametric and non-parametric continuous data were analyzed using a Student’s *t*-test or Mann–Whitney U test. Discrete data were analyzed using a χ^2^ test. Data were analyzed using Excel^®^ or third-party statistical calculation software. Estimated power calculations were based on a similar study by Fukuda and colleagues where results showed a 46% reduction in days of therapy for chosen antibiotics [20]. The Fukuda study is similar to this study as it was done in a community hospital setting and also aimed to assess the impact of a pharmacist-led antimicrobial stewardship program on the number of days of antimicrobial therapy. A key difference to note is that the Fukuda study mainly focused on patients with gram-negative bacteremia. Based on modeling from a sample estimate from the Fukuda study, it was estimated that a sample size of 406 would be needed to show appropriate power at 0.8 with a reduction of 3% in days of therapy [20]. This modest reduction was chosen due to the variability in the treatment of 2019 coronavirus disease (COVID-19) patients as well as the difference in the antibiotics evaluated in the Fukuda and colleagues study versus those evaluated in this study [20].

## 3. Results

**Figure 1:**
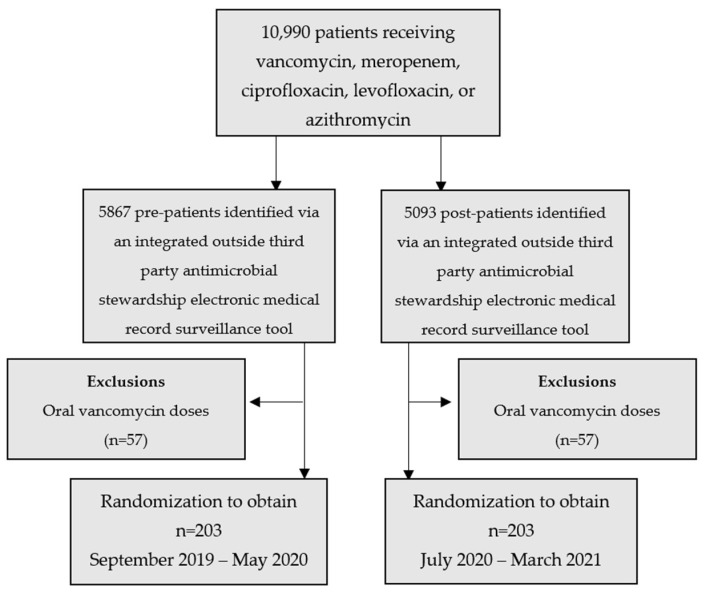
Study population.

As seen in Figure 1, 10,990 patients were initially identified to have received vancomycin, meropenem, ciprofloxacin, levofloxacin, or azithromycin. These patients were divided into the pre-intervention and post-intervention groups via an integrated outside third-party antimicrobial stewardship electronic medical record surveillance tool which identified pre-intervention with 5867 patients and post-intervention with 5091. Of those identified, 332 of those patients were excluded. The most common reason for exclusion was surgical prophylaxis which excluded 91 patients in the pre- (1.55%) and post- (1.77%) intervention groups. After exclusions were performed, the pre- and post-intervention groups were then identified via a randomization calculator to obtain a total of 406 patients.

The patient characteristics of the pre- and post-intervention groups were similar except for age, hospital service, and allergies. (Table 1). The mean age was 67 years ± 18 for the pre-intervention group and 62 years ± 21 for the post-intervention group (*p* = 0.0271). The most common hospital service in which patients were admitted was the general medicine unit, with 29% of all admissions in the pre-intervention group and 22% of all admissions in the post-intervention group. Allergy documentation for no known drug allergies (NKDA) was documented in 148 patient profiles (73%) in the pre-intervention group and in 169 patient profiles (83%) in the post-intervention group (*p* = 0.0223).

### 3.1. Primary Outcome

Antimicrobial days of therapy for vancomycin, azithromycin, levofloxacin, ciprofloxacin, and meropenem were reduced in the post-intervention period. An average of 3.47 ± 2.46 days (pre-intervention, *n* = 203) and 3.21 ± 2.52 days (post-intervention, *n* = 203) were observed for this outcome. This was a reduction of 9.11% (*p* < 0.0001) when comparing the pre- and post-intervention periods.

### 3.2. Secondary Outcomes

Pharmacists performed 75 interventions pre-intervention and 102 interventions post-intervention (*p* = 0.0092). The cost avoidance secondary outcome was $15,138 (pre-intervention, *n* = 203) and $21,103 (post-intervention, *n* = 203) (*p* = 0.5859).

## 4. Discussion

In this single center retrospective study, it was found that the expansion of prospective audit and feedback beyond the antimicrobial stewardship clinical pharmacy specialist to the pharmacy department had an impact on decreasing antimicrobial days of therapy through implementation and/or education provided via policies, procedures, in-services, presentations, etc. The decrease in antimicrobial days of therapy was a modest reduction of 9.11% between the pre- and post-intervention groups. The modest reduction was speculated to be connected to the main limitation of assessing the post-intervention group during the COVID-19 pandemic where variability in antibiotic usage and changing recommendations on how to treat COVID-19 patients occurred [19,20,21,22]. Another possibility in which the percentage difference was low may be due to some interventions involving solely education and not required policies or procedures performed by the pharmacy department. In addition to that, the impact of education on providers is unknown and could have affected antibiotic ordering, pharmacist recommendations, and documented interventions. Only education was performed on ciprofloxacin, levofloxacin, and meropenem, and the restriction criteria for these were not yet implemented, which could have also contributed to the decreased percentage difference in antibiotic use as these were not yet related to required policies or procedures.

It was interesting that only one secondary outcome reached statistical significance despite the cost avoidance being related to the outcomes documented by the pharmacy team. It is hypothesized that this is likely due to each intervention being correlated to a specific pre-determined cost avoidance number; therefore, although the number of interventions increased, those interventions may have been connected to a lower overall cost avoidance. The difference in cost avoidance could have also been due to the change in interventions that were documented. The cost avoidance numbers are connected to the interventions that the pharmacist documents in the antimicrobial stewardship electronic medical record surveillance tool. Due to the change in vancomycin becoming pharmacy-to-dose, the interventions that were documented changed.

This study had several limitations. It is hypothesized that the COVID-19 pandemic may have negatively skewed results due to the overuse of antimicrobials and a lack of guidelines on COVID-19 treatment [19,20,21,22]. It is also hypothesized that the overwhelming requirements (increased time, increased number of patients, etc.) of treating COVID-19 patients could have skewed the number of interventions that were documented or performed by the pharmacy department. Another noted limitation includes the transition from the pharmacy receiving consultations to dose vancomycin to the pharmacy automatically being consulted to dose all vancomycin patients in the post-intervention period. Consequently, this prompted a change in consultation documentation which resulted in the pre-intervention group performing more documentations of their interventions related to vancomycin versus the post-intervention group in which vancomycin became pharmacy-to-dose. This resulted in the removal of required documented interventions as it was automatically assumed that a pharmacist would be dosing and monitoring those medications. The emergency medicine department automatic dispensing cabinets (ADCs) were placed on override which could increase the use of antibiotics that were included in this study due to no prospective pharmacist verification of orders. Interventional documentation on learned education was not standardized among the pharmacy department and was voluntarily documented. The quantification of provider practices based on education to the pharmacy department to providers they interact with cannot be determined. This study was also a single center retrospective chart review which may decrease generalizability to other hospitals and/or academic medical centers that differ in practices, pharmacist department size, antimicrobial stewardship program size, patient population, and number of patients admitted.

## 5. Conclusions

The implementation of a dedicated antimicrobial stewardship clinical pharmacist responsible for developing policies, procedures, and education successfully reduced antimicrobial days of therapy and increased documented interventions. The extension of the antimicrobial stewardship program to the pharmacy department through policies, procedures, and education can benefit antibiotic use and cost avoidance. Pharmacists have an important role in antimicrobial stewardship to help combat the increase in antimicrobial resistance worldwide, whether their role is mainly dedicated to infectious diseases or not.

We hope that the publication of this study increases evidentiary support of the impact of antimicrobial stewardship pharmacists and allows for the further justification of future positions.

## Figures and Tables

**Table 1:** Demographic and characteristics of adults.

Demographics	Pre-Intervention *n* = 203	Post-Intervention *n* = 203	*p*-Value
**Age, years**			*p* = 0.0271
Mean (SD)	67 (18)	62 (21)	
**Length of stay, days**			*p* = 0.05892
Median (IQR)	4.02 (2.19–7.55)	4.28 (2.10–8.09)	
**Male, *n* (%)**			*p* = 0.04845
Male	104 (51)	111 (55)	
**Hospital Service, *n* (%)**			*p* < 0.0001
General medicine	59 (29)	45 (22)	
Oncology	48 (24)	37 (18)	
Surgical care	11 (5)	21 (10)	
Intermediate care	11 (5)	10 (5)	
Neurointensive care	4 (2)	5 (3)	
**Allergies to antibiotics, *n* (%)**			*p* = 0.0223
No known drug allergies (NKDA)	148 (73)	169 (83)	
Allergies with no listed reaction	46 (23)	25 (12)	
Allergies with listed reactions	9 (4)	9 (4)	
**Antibiotics, *n* (%)**			*p* = 0.8459
Vancomycin	101 (50)	99 (49)	
Azithromycin	49 (24)	55 (27)	
Ciprofloxacin	23 (11)	21 (10)	
Levofloxacin	16 (8)	16 (8)	
Meropenem	13 (6)	10 (5)	

## Data Availability

Please contact corresponding author for questions regarding data.
